# Correlation of cell-free DNA plasma concentration with severity of non-alcoholic fatty liver disease

**DOI:** 10.1186/s12967-017-1208-6

**Published:** 2017-05-19

**Authors:** Thomas Karlas, Lara Weise, Stephanie Kuhn, Felix Krenzien, Matthias Mehdorn, David Petroff, Nicolas Linder, Alexander Schaudinn, Harald Busse, Volker Keim, Johann Pratschke, Johannes Wiegand, Katrin Splith, Moritz Schmelzle

**Affiliations:** 1 Department of Medicine, Neurology and Dermatology, Division of Gastroenterology and Rheumatology, University Hospital Leipzig, Leipzig, Germany; 2 IFB Adiposity Diseases, Leipzig University Medical Center, Leipzig, Germany; 3 Medical Faculty, Leipzig University, Leipzig, Germany; 4 Department Environmental Immunology, Helmholtz Centre for Environmental Research GmbH-UFZ, Leipzig, Germany; 5 Department of Surgery, Campus Virchow-Klinikum, Charité-Universitätsmedizin Berlin, Berlin, Germany; 6 Department of Visceral-, Transplantation-, Thoracic- and Vascular Surgery, University Hospital Leipzig, Leipzig, Germany; 7 Clinical Trial Centre, Leipzig University, Leipzig, Germany; 8 Department of Diagnostic and Interventional Radiology, University Hospital Leipzig, Leipzig, Germany

**Keywords:** Controlled attenuation parameter, Transient elastography, Cell-free DNA, MR-spectroscopy, Non-alcoholic fatty liver disease, Non-alcoholic steatohepatitis

## Abstract

**Background:**

The assessment of fibrosis and inflammatory activity is essential to identify patients with non-alcoholic fatty liver disease (NAFLD) at risk for progressive disease. Serum markers and ultrasound-based methods can replace liver biopsy for fibrosis staging, whereas non-invasive characterization of inflammatory activity remains a clinical challenge. Cell-free DNA (cfDNA) is a novel non-invasive biomarker for assessing cellular inflammation and cell death, which has not been evaluated in NAFLD.

**Methods:**

Patients and healthy controls from two previous studies were included. NAFLD disease activity and severity were non-invasively characterized by liver stiffness measurement (transient elastography, TE) including steatosis assessment with controlled attenuation parameter (CAP), single-proton magnetic resonance spectroscopy (^1^H-MRS) for determination of hepatic fat fraction, aminotransferases and serum ferritin. cfDNA levels (90 and 222 bp fragments) were analyzed using quantitative real-time PCR.

**Results:**

Fifty-eight NAFLD patients (age 62 ± 11 years, BMI 28.2 ± 3.5 kg/m^2^) and 13 healthy controls (age 38 ± 12 years, BMI 22.4 ± 2.1 kg/m^2^) were included. 90 bp cfDNA levels were significantly higher in NAFLD patients compared to healthy controls: 3.7 (1.3–23.1) vs. 2.9 (1.4–4.1) ng/mL (p = 0.014). In the NAFLD cohort, circulating cfDNA correlated significantly with disease activity and severity, especially in patients with elevated liver stiffness (n = 13, 22%) compared to cases with TE values ≤7 kPa: cf90 bp 6.05 (2.41–23.13) vs. 3.16 (1.29–7.31) ng/mL (p < 0.001), and cf222 bp 14.41 (9.27–22.90) vs. 11.32 (6.05–18.28) ng/mL (p = 0.0041).

**Conclusions:**

Cell-free DNA plasma concentration correlates with established non-invasive markers of NAFLD activity and severity. Therefore, cfDNA should be further evaluated as biomarker for identifying patients at risk for progressive NAFLD.

**Electronic supplementary material:**

The online version of this article (doi:10.1186/s12967-017-1208-6) contains supplementary material, which is available to authorized users.

## Background

Non-alcoholic fatty liver disease (NAFLD) is an emerging worldwide epidemic, which will replace viral hepatitis as major cause of hepatic mortality within the next decades [1]. The spectrum of NAFLD comprises both simple steatosis and non-alcoholic steatohepatitis (NASH): while simple steatosis has a low risk of disease progression, a relevant proportion of NASH patients will develop fibrosis and ultimately cirrhosis [2]. In this regard, presence of fibrosis is the strongest predictor for liver related morbidity and mortality [3].

Traditionally, liver biopsy is considered as the gold standard method for the diagnosis and fibrosis staging of NAFLD [4]. However, due to potential complications and diagnostic limitations (i.e. sampling errors and restricted repeatability), various non-invasive techniques for diagnosis and estimation of disease severity have been proposed [5]. Among them, ultrasound-based methods for assessment of liver stiffness (elastography) have shown high accuracy in discriminating patients at risk for advanced fibrosis and impaired prognosis in different chronic liver diseases including NAFLD [6]. Recent advancements of elastography methods allow the characterization of liver steatosis simultaneously with liver stiffness measurement (LSM), e.g. with the controlled attenuation parameter (CAP) software [7]. However, while non-invasive estimation of fibrosis and liver steatosis has already been implemented in clinical practice, serum-based biomarkers for the presence of NASH such as liver enzymes and ferritin show limited accuracy, although they are widely used in clinical practice [4, 5]. Hence, the precise characterization of hepatic inflammatory activity still relies on histological assessment, which is a major drawback for diagnosis of NASH in clinical practice [1].

Facing the increasing prevalence of NAFLD, there is an urgent need to identify patients with NASH at risk for progressive disease with non-invasive markers that can be used as a point-of care approach [5]. In this line, several laboratory and ultrasound based markers have been approved for estimation of fibrosis severity, whereas non-invasive characterization of hepatic inflammation remains a diagnostic challenge [1, 6]. Cell-free plasma DNA (cfDNA) consists of small nucleic acid fragments originating from destructed cells that circulate in the blood stream. cfDNA levels correlate with disease stage and severity of tissue damage in several clinical conditions [8], especially in patients with malignant diseases where it may represent a sensitive approach for diagnosis of occult tumors and metastases [9, 10]. Studies on oxidative stress and inflammatory response suggest a potential diagnostic value of cfDNA in liver disease as well [11]. Particularly due to its short half-life of minutes to hours cfDNA might provide a snapshot of current disease activity [9]. However, the diagnostic value of cfDNA has not yet been studied in NAFLD patients. We therefore analyzed the correlation of cfDNA with extent and severity of NAFLD.

## Methods

### Patients and controls

**Fig. 1:**
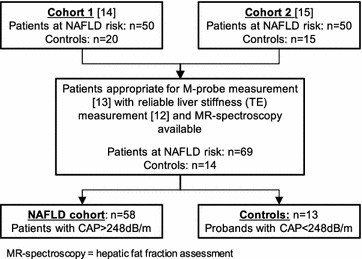
Definition of the patient cohort. *NAFLD* non-alcoholic fatty liver disease, *CAP* controlled attenuation parameter, *TE* transient elastography, *MR* magnetic resonance

For the present analysis, blood samples from two previous published cohorts have been analyzed (Fig. 1). From the initial cohorts, only patients with reliable liver stiffness measurement (LSM) [12], single-proton magnetic resonance spectroscopy (^1^H-MRS) [13] and elevated hepatic fat content defined by CAP ≥ 248 dB/m [7] were included in final analysis.

### Ultrasound-bases liver stiffness and steatosis assessment

Liver stiffness was measured using transient elastography (TE) according to the manufacturer’s recommendation as described before [14, 15]: In brief, all participants underwent conventional ultrasound to rule out mechanical cholestasis or congestive liver disease. Skin-to-liver-capsule distance at the TE measuring site was recorded with a high frequency linear transducer. For the present analysis, only subjects eligible for TE M-probe with an skin-to-liver-capsule distance ≤25 mm were included. Cases with fewer than 10 valid measurements or an interquartile range (IQR) >30% of the median LSM value (only in cases with liver stiffness ≥7.1 kPa) were excluded. According to Wong et al. [16], LSM values >7.0 kPa indicated risk of fibrosis and LSM values >9.6 kPa defined a high risk of advanced fibrosis.

Controlled attenuation parameter is a measure of TE ultrasonic signal attenuation and was computed simultaneously during LSM using the M-probe [14, 15]. CAP values ≥248 dB/m defined presence of hepatic steatosis. CAP values >282 dB/m indicated advanced steatosis [7].

### Magnetic resonance spectroscopy and volumetry

Patients underwent MRI examination at 1.5 T according to a previously described protocol [14, 15]. Single-voxel proton magnetic resonance spectroscopy (^1^H-MRS) was used to assess the hepatic lipid components. In short, voxels (20 × 20 × 20 mm^3^) were placed in the right liver lobe avoiding bile ducts and larger vessels. Relative lipid concentrations were measured with a commercial MRS analysis tool (LCModel, Oakville, Canada). Spectroscopic peak areas of water and fat were corrected for T2 relaxation effects and used to calculate the hepatic fat fraction (in %) [14, 15].

### Laboratory assessment

Routine liver function tests were available from the original study databases. None of the patients had alanine aminotransferase (ALT) or aspartate aminotransferase (AST) levels >5× upper limit of normal (ULN), as defined by the initial study protocols [14, 15].

In addition, stored ethylenediaminetetraacetic acid (EDTA) blood samples (centrifuged at 1000*g* for 10 min within 2 h after collection; plasma layer carefully transferred to a new vial and stored at −20 °C) were available and used for deoxyribonucleic acid (DNA) extraction.

### DNA extraction

After equilibrating probes to room temperature, plasma was centrifuged at 1000*g* for 10 min to remove any possible remaining cell components.

Cell-free DNA was extracted from 200 µL plasma using the QIAamp Blood DNA Mini kit (Qiagen) according to the manufacture’s instructions (spin protocol). To increase the yield of DNA the time of incubation with AE buffer was prolonged from 5 min to 10 min. The DNA was eluted in 50 µL AE buffer and stored at −20 °C for at least 24 h before polymerase chain reaction (PCR) analysis.

### PCR analysis

Cell-free plasma DNA was quantified by real time PCR (qPCR) using the 7500 Real Time PCR System (Applied Biosystems by Life Technologies,). Two sets of primers were used amplifying a 90 and a 222 bp fragment (cf90, cf222), respectively. Both fragments were amplified using the same forward primer (5′-TGCCGCAATAAACATACGTG-3′) and a different reverse primer (cf90: 5′-GACCCAGCCATCCCATTAC-3′, cf222: 5′-AACAACAGGTGCTGGAGAGG-3′). Both fragments are found on the L1PA2 element which is a LINE sequence. LINEs (long-interspearsed nulear elements) are non-coding DNA which show thousands of repeats in the human genome. Primers for both LINE1 fragments were taken from the literature [17]. Reactions were set up in a total volume of 20 µL including 10 µL GoTaq^®^ Mastermix (Promega), 8 µL nuclease free water, 0.5 µL forward primer (10 µM), 0.5 µL reverse primer (10 µM) and 1 µL of extracted DNA eluted in AE buffer. Cycling conditions were set up as recommended by the producer: initial activation for 2 min at 95 °C and 40 cycles of denaturation at 95 °C for 15 s and annealing and extension first at 55 °C for 30 s, subsequently heated up to 60 °C for 1 min. Final extension followed at 72 °C for 5 min and then the plate was cooled down to 4 °C. Non-template controls were included on each PCR plate to confirm the absence of contamination. The efficiency and specificity of the primers was previously described [17, 18]. The qPCR results were analyzed using the 7500 Software (v2.0.6).

To quantify the cfDNA a standard curve was generated. Therefore, blood was taken from a healthy volunteer and the yield of total DNA extracted from 200 µL whole blood with QIAmp Blood DNA Mini kit as described above. A PCR was performed from the eluate with the GoTaq^®^ qPCR Master Mix (Promega) to obtain the respective fragment and concentration was measured using the Nanodrop 2000 (Thermo Fisher Scientific Inc.). The PCR-product was cleaned up from spare nucleotides and primers by Rapid PCR clean up enzyme set (New England BioLabs GmbH), then concentration was measured with the Nanodrop 2000 and a dilution series was made (1; 1:10; 1:100; 1:500; 1:1000; 1:2000; 1:4000; 1:8000; 1:16,000; 1:32,000; 1:64,000; 1:128,000; 1:256,000; 1:512,000). For each dilution a threshold cycle was determined and at least three dilutions of the following (1:16,000; 1:32,000; 1:64,000; 1:128,000; 1:256,000; 1:512,000) were measured in every reaction plate. The threshold cycles for each dilution were used to generate a standard curve for the determination of cfDNA yield in every probe.

### DNA integrity index analysis

The DNA integrity index is a measure of the ratio of short and long cfDNA fragments and provides information on the origin of cfDNA, since smaller fragments are primarily released in apoptosis, while the release of larger fragments has mainly been described in mechanisms of uncontrolled cell death [19, 20]. DNA used for the standard curve was used as reference to determine the relative DNA strand integrity in plasma DNA. ∆Ct222 was calculated subtracting the Ct value for the 222 bp fragment of a sample from the reference. Also, the ∆Ct90 was calculated with the Ct value for the 90 bp fragment of a sample. To obtain a ∆∆Ct value the ∆Ct value for 222 bp was subtracted from the ∆Ct value for the 90 bp. The integrity index was calculated for each probe as exponential of (−∆∆Ct × LN 2) as described before [19, 20].

### Statistical analysis

Statistical analyses were performed using commercial software Prism 6 (GraphPad Software, La Jolla, CA, USA) and R version 3.3 with the lme4 package [21]. Categorical variables are expressed as frequencies and percentages; continuous variables were expressed either as mean ± standard deviation or median and range, as appropriate. Non-parametric tests (Mann–Whitney U test, Kruskal–Wallis test with post hoc analysis using the Dunn procedure) were used for comparison of independent samples. For mean values, the *t* test was applied. Multivariate analyses were performed using linear mixed models where PCR batch was taken as a random effect. Model selection was based on Akaike’s Information Criterion (AIC). Pearson correlation coefficients were calculated to analyze the degree of association between two variables. For determination of optimal diagnostic cut-offs, receiver operating characteristics (ROC) analysis by maximizing the Youden index were performed. Statistical significance was defined as p < 0.05.

## Results

### Clinical characteristics of the study cohorts

58 NAFLD patients and 13 healthy controls matched the inclusion criteria and were eligible for the analysis (Fig. 1). Clinical characteristics of the study cohort are assorted in Table 1.

**Table 1:** Baseline characteristics of the study cohort

	Controls n = 13	NAFLD cohort n = 58
Anthropometry		
Sex (male/female)	6 (46.2%)/7 (53.8%)	32 (55.2%)/26 (44.8%)
Age (years^a^)	37.7 ± 11.5	62.1 ± 11.0
BMI (kg/m^2a^)	22.4 ± 2.1	28.2 ± 3.5
<25 (n)	11 (84.6%)	12 (20.7%)
25–30 (n)	2 (15.4%)	30 (51.7%)
30–35 (n)	0 (0%)	15 (25.9%)
>35 (n)	0 (0%)	1 (1.7%)
Waist-to-hip (ratio^a^)	0.87 ± 0.14	0.96 ± 0.09
Diabetes (n)	0 (0%)	37 (63.8%)
Laboratory values		
HbA1c (%^b^)	5.0 (4.7–5.4)	5.7 (4.6–8.0)
ALT/ULN (ratio^b^)	0.4 (0.3–0.6)	0.9 (0.3–3.3)
AST/ULN (ratio^b^)	0.5 (0.4–0.7)	0.8 (0.3–2.1)
Ferritin/ULN (ratio^b^)	0.3 (0.05–2.0)	0.7 (0.1–5.5)
Non-invasive liver assessment		
^1^H-MRS (rHLC^b^)	0.8 (0–8.6)	12.8 (1.2–41.1)
CAP (dB/m^b^)	210 (100–231)	310 (249–397)
Liver stiffness (kPa^b^)	4.4 (2.3–5.9)	5.3 (1.9–70.6)

*BMI* body mass index, *HbA1c* hemoglobin A1c, *ALT* alanine aminotransferase, *AST* aspartate aminotransferase, *ULN* upper limit of normal, ^*1*^ *H*-*MRS* single-proton magnetic resonance spectroscopy, *CAP* controlled attenuation parameter, *rHLC* relative hepatic lipid content

^a^ Values presented as mean and standard deviation

^b^ Values presented as median and range

NAFLD patients were characterized by higher age, higher BMI, and higher WHR values compared to the healthy controls (p < 0.0001, respectively) and showed a high prevalence of features of the metabolic syndrome: overweight (BMI > 25 kg/m^2^) 79%; obesity (BMI > 30 kg/m^2^) 28%; type 2 diabetes 64%).

Serum ALT and AST levels were significantly higher in NAFLD patients than in healthy controls (p < 0.0001, respectively). In the NAFLD cohort, elevated ALT and AST were observed in 26 and 18 cases (45 and 31%, respectively). In 12 cases (21%) both values were increased concurrently. Seventeen patients (30%) had elevated serum ferritin levels.

Because of the substantial differences between the cohorts, the primary analyses were performed in the NAFLD cohort alone.

### Liver stiffness and liver steatosis measurement

All healthy controls had normal LSM values as defined by the protocol (Table 1). Among NAFLD patients, 13 (22%) had increased LSM values over 7.0 kPa indicative of inflammation and fibrosis. In these patients, 10 (17%) had LSM values higher than 9.6 kPa indicating high risk of advanced fibrosis.

Controlled attenuation parameter classified NAFLD patients as follows [7]: mild steatosis (CAP ≤ 268 dB/m) n = 7 (12%), moderate steatosis (CAP 269–282 dB/m) n = 9 (16%), and advanced steatosis (CAP > 282 dB/m) n = 42 cases (72%), respectively.

MR spectroscopy showed a significant difference in hepatic fat between healthy controls and NAFLD patients. Of these, 33 patients (57%) had relative hepatic lipid content values over 10%.

In NAFLD patients, ^1^H-MRS and CAP values were correlated (r = 0.466), whereas liver stiffness did not correlate with either CAP (r = 0.036), or ^1^H-MRS (r = 0.063).

### Association of cfDNA with anthropometry and factors of the metabolic syndrome

The cf90 fragment concentrations were increased in the NAFLD cohort compared to the healthy controls: 3.7 (1.3–23.1) vs. 2.9 (1.4–4.1) ng/mL, (p = 0.014). There was a similar tendency for cf222: 11.54 (6.1–22.9) vs. 10.8 (6.4–15.0) ng/mL (p = 0.054).

In the NAFLD cohort, no significant association of cfDNA (both fragments) with age, BMI or presence of type 2 diabetes was detected in a linear mixed model, but there was a dependence on sex in the case of cf222 (2.4 ng/mL lower for men, p = 0.0051). The model without age, BMI and type 2 diabetes had a lower AIC, so that we retained only sex for further analyses. To have a consistent model, sex was also used for cf90.

### Association of cfDNA with NAFLD severity

NAFLD patients with increased liver stiffness (>7.0 kPa) had significantly higher cf90 and cf222 fragment concentrations. Those at risk for advanced fibrosis (>9.6 kPa) showed elevated levels of cf90 fragment concentrations and there are indications for a similar effect with cf222. Cf90 fragment concentrations were significantly higher in NAFLD patients with increased AST values, while no such difference was observed for ALT values. Cf222 fragments were not significantly associated with either ALT or AST. Significant associations were seen between ferritin and both cfDNA fragments. Neither CAP nor ^1^H-MRS showed significant associations with concentration of cfDNA. Accordingly, no differences in cfDNA concentrations between NAFLD patients with and without advanced steatosis were observed (Table 2).

**Table 2:** Association of cfDNA levels with features of disease severity in NAFLD patients

Parameter	cfDNA fragment	Median (range)		p value
*Liver stiffness*		*≤7.0 kPa (n = 45)*	*>7.0 kPa (n = 13)*	
	90 bp	3.16 (1.29–7.31)	6.05 (2.41–23.13)	<0.001
	222 bp	11.32 (6.05–18.28)	14.41 (9.27–22.90)	0.0041
		*≤9.6 kPa (n = 48)*	*>9.6 kPa (n = 10)*	
	90 bp	3.22 (1.29–8.17)	5.78 (2.41–23.13)	0.011
	222 bp	11.35 (6.05–19.74)	14.27 (9.27–22.90)	0.059
*Inflammatory activity*		*ALT ≤ ULN (n = 32)*	*ALT > ULN (n = 26)*	
	90 bp	3.08 (1.29–23.13)	3.93 (2.19–8.62)	0.26
	222 bp	11.20 (6.92–22.90)	12.89 (6.05–19.74)	0.23
		*AST ≤ ULN (n = 40)*	*AST > ULN (n = 18)*	
	90 bp	3.05 (1.29–8.17)	5.18 (1.38–23.13)	0.038
	222 bp	11.32 (6.05–19.74)	13.79 (8.66–22.90)	0.14
		*Ferritin ≤ ULN (n = 40)*	*Ferritin > ULN (n = 17)*	
	90 bp	3.21 (1.29–8.17)	4.70 (2.19–23.13)	0.026
	222 bp	11.20 (6.05–19.74)	13.25 (10.0–22.90)	0.0071
*Steatosis*		*CAP ≤ 282 dB/m (n = 16)*	*CAP > 282 dB/m (n = 42)*	
	90 bp	3.51 (1.38–7.56)	3.71 (1.29–23.13)	0.12
	222 bp	12.00 (6.05–18.28)	11.49 (6.91–22.90)	0.083
		*Fat fraction*	*Fat fraction*	
		(^*1*^ *H-MRS) ≤10% (n = 25)*	(^*1*^ *H-MRS) >10% (n = 33)*	
	90 bp	2.97 (1.28–8.62)	3.90 (1.38–23.13)	0.13
	222 bp	11.35 (6.92–18.28)	12.35 (6.05–22.90)	0.34

Values presented as median and range (ng/mL), p-values are taken from a linear mixed model with sex as a covariate and PCR-batch as a random term

^*1*^ *H*-*MRS* single-proton magnetic resonance spectroscopy, *CAP* controlled attenuation parameter, *rHLC* relative hepatic lipid content, *ALT* alanine aminotransferase, *AST* aspartate aminotransferase, *ULN* upper limit of normal

To avoid collinearity, multivariate models considered only one measure of inflammatory activity and one of steatosis (fat fraction, which is less related to liver stiffness than CAP). These corroborated the findings in Table 2, except that AST was no longer associated with cf90 upon taking liver stiffness and fat fraction into account (with sex as a covariate).

Based on these findings, we classified our NAFLD cohort according to disease severity:

(i) NAFLD patients with normal liver enzymes and normal liver stiffness (“pure steatosis”),
(ii) patients with elevated liver enzymes (ALT and/or AST > ULN) and liver stiffness ≤7.0 kPa,
(iii) patients with elevated liver stiffness >7.0 kPa. All of them had elevated ALT and/or AST.

**Fig. 2:**
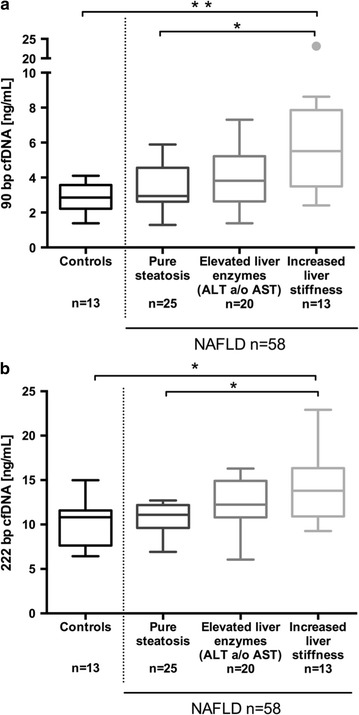
Association of cell-free DNA with NAFLD severity. Serum levels of both (**a**) 90 bp (*p* = 0.001) and (**b**) 222 bp (*p* = 0.009) cfDNA fragments correlate significantly with NAFLD severity (Kruskal–Wallis test and post hoc analysis, *Asterisk* indicates *p* values <0.05; *double asterisk* indicates *p* values <0.01). *cfDNA* cell-free DNA, *NAFLD* non-alcoholic fatty liver disease, *ALT* alanine aminotransferase, *AST* aspartate aminotransferase

Plasma levels of both cfDNA fragments correlated with this classification (Fig. 2).

Thus, it is natural to question if cfDNA has clinical potential to help in identifying patients at risk for hepatic fibrosis, defined by elevated LSM. Areas under the curve from receiving operating characteristic were 0.78 (95% CI 0.63–0.93) for cf90 and 0.74 (95% CI 0.58–0.90) for cf222. The optimal cut-off for the former was 5.5 ng/mL (specificity 0.93, sensitivity 0.54) and 12.8 ng/mL (specifity 0.77, sensitivity 0.69).

### cfDNA integrity index

Using a linear mixed model with sex as a covariate and PCR batch as a random effect, the integrity index was not found to differ significantly between NAFLD patients and the control group (p = 0.52). In the NAFLD cohort, no association of the cfDNA integrity index with elevated aminotransferase levels, liver stiffness risk categories or advanced steatosis (defined in Table 2) could be observed (all p values >0.14).

## Discussion

In light of increasing NAFLD prevalence, non-invasive markers for the evaluation of disease severity are needed for decision making in clinical practice [6]. The progression of NAFLD is driven by inflammatory mechanisms involving apoptosis and necrosis of hepatocytes, which ultimately result in the development of liver fibrosis and cirrhosis [22–25]. In the course of these inflammatory processes, DNA is released into the bloodstream. CfDNA has recently been reported as promising biomarker for risk and outcome prediction in a variety of diseases including cancer [10], organ damage [26] as well as acute graft rejection after solid organ transplantation [27–29].

We here demonstrate an association of circulating cfDNA fragments with established non-invasive markers of NAFLD severity, especially in patients with increased liver stiffness and elevated AST and ferritin levels, who are at high risk of ongoing NASH and of disease progression [5, 6, 30]. Elevation of serum aminotransferases, especially AST, and increased ferritin indicate oxidative stress and inflammatory activity in NAFLD [30–33]. The association of cf90 and cf222 with these markers probably reflects hepatic cfDNA release induced by inflammation and cell death, i.e. high NASH activity [4]. Moreover, further cellular systems involved in NASH pathogenesis, e.g. visceral fat, may contribute to the increase of cfDNA. Nishimoto and co-workers [34] recently linked adipose tissue inflammation to cfDNA released by adipocytes. Similar signaling mechanisms may play a role in NASH progression. A pro-inflammatory effect of cfDNA can be mediated through the secretion of TNF-α [35] and activation of Toll-like receptors [36, 37]. Future studies now need to clarify the origin of the cfDNA in patients with NASH and should also analyze its potential role as a modulator of inflammatory activity. Furthermore, longitudinal observations should address the kinetics of cfDNA release in the course of NAFLD, which might be associated with the ratio of long to short cfDNA fragments [38]. The cfDNA integrity index, which was not associated with diseases severity in our cross-sectional analysis, may potentially provide additional information on dynamics of disease activity.

The analysis of cfDNA only requires a blood sample. Morover, short half-life makes this parameter interesting for monitoring disease activity even in short term. CfDNA is already clinically relevant in prenatal testing [39], whereas its diagnostic role in the context of chronic liver diseases still needs to be defined. Sensitivity and specificity of cfDNA levels for detection of advanced NAFLD are limited in our cohort and biopsy-controlled large-scale studies are needed to assess its definite diagnostic accuracy. However, the combination of cfDNA with complementary non-invasive methods may provide higher accuracy for detection of NASH activity and significant fibrosis. In addition, circulating DNA markers may provide an alternative for patients with contraindications to TE and MRI, such as morbid obesity [40]. Furthermore, circulating donor cfDNA has been suggested as a marker for graft injury in liver transplant recipients [29].

Future research directions should consider epigenetic markers such as cfDNA methylation signatures to identify tissue-specific cell death [41]. Along these lines, recent diagnostic studies on cfDNA in patients with malignant diseases have studied tumor specific DNA fragments, which performed better than total plasma cfDNA [9]. In the liver setting, Hardy et al. have demonstrated a higher methylation density on circulating DNA in NAFLD patients compared to controls, and methylation density was even higher in advanced than in mild fibrosis [42]. Therefore, the assessment of liver specific methylation may increase the diagnostic value of cfDNA measurement and should be incorporated in future study protocols.

This study has several limitations: Our findings rely on secondary analysis of data obtained for previous research. Therefore, the case number is limited and associations with further markers of oxidative stress and inflammation could not be studied. In addition, the non-invasive reference standards liver stiffness and CAP measurement as well as laboratory tests and ^1^H-MRS are only surrogates for NAFLD activity and severity with limited accuracy in individual cases, and cannot completely replace histological staging and grading [6]. However, we believe that due to the invasive nature of liver biopsy, which is usually restricted to patients with indeterminate diagnosis in clinical practise, a pilot study with non-invasive surrogates is appropriate as a first approach. The methods used here, namely liver stiffness measurement with TE, have been approved as a biopsy alternative for fibrosis staging and correlate with prognosis in various chronic liver diseases [6]. However, we underline that our findings now require verification in prospective biopsy-controlled studies, which should focus on inflammatory activity and hepatocyte apoptosis.

## Conclusion

Cell-free plasma DNA plasma concentration correlates with established non-invasive markers of NAFLD activity and severity, and thus represents an interesting new biomarker for assessing NAFLD. Future studies should analyse the role of cfDNA as a mediator of tissue inflammation, and should focus on its diagnostic value for discriminating patients at risk for NAFLD related morbidity and mortality. Long-term follow-up is warranted to assess the correlation between cfDNA and disease progression.

## Additional file

**Additional file 1.** Table clinical dataset.

## Abbreviations

NAFLD: non-alcoholic fatty liver disease
cfDNA: cell-free DNA
TE: transient elastography
CAP: controlled attenuation parameter
^1^H-MRS: single-proton magnetic resonance spectroscopy
BMI: body mass index
ALT: alanine aminotransferase
AST: aspartate aminotransferase
NASH: non-alcoholic steatohepatitis
LSM: liver stiffness measurement
IQR: interquartile range
ULN: upper limit of normal
EDTA: ethylenediaminetetraacetic acid
DNA: deoxyribonucleic acid
PCR: polymerase chain reaction
LINE: long-interspearsed nuclear element
AIC: Akaike’s Information Criterion
ROC: receiver operating characteristics
HbA1c: hemoglobin A1c
rHLC: relative hepatic lipid content

## References

[1] EASL-EASD-EASO (2016). Clinical practice guidelines for the management of non-alcoholic fatty liver disease. J Hepatol.

[2] Singh S, Allen AM, Wang Z, Prokop LJ, Murad MH, Loomba R (2015). Fibrosis progression in nonalcoholic fatty liver vs nonalcoholic steatohepatitis: a systematic review and meta-analysis of paired-biopsy studies. Clin Gastroenterol Hepatol.

[3] Ekstedt M, Hagström H, Nasr P, Fredrikson M, Stål P, Kechagias S, Hultcrantz R (2015). Fibrosis stage is the strongest predictor for disease-specific mortality in NAFLD after up to 33 years of follow-up. Hepatology.

[4] Bedossa P (2016). Histological assessment of NAFLD. Dig Dis Sci.

[5] Dyson JK, McPherson S, Anstee QM (2013). Non-alcoholic fatty liver disease: non-invasive investigation and risk stratification. J Clin Pathol.

[6] EASL-ALEH (2015). Clinical practice guidelines: non-invasive tests for evaluation of liver disease severity and prognosis. J Hepatol.

[7] Karlas T, Petroff D, Sasso M, Fan JG, Mi YQ, de Lédinghen V, Kumar M, Lupsor-Platon M, Han KH, Cardoso AC, Ferraioli G, Chan WK, Wong VW, Myers RP, Chayama K, Friedrich-Rust M, Beaugrand M, Shen F, Hiriart JB, Sarin SK, Badea R, Jung KS, Marcellin P, Filice C, Mahadeva S, Wong GL, Crotty P, Masaki K, Bojunga J, Bedossa P, Keim V, Wiegand J (2017). Individual patient data meta-analysis of controlled attenuation parameter (CAP) technology for assessing steatosis. J Hepatol.

[8] Swarup V, Rajeswari MR (2007). Circulating (cell-free) nucleic acids–a promising, non-invasive tool for early detection of several human diseases. FEBS Lett.

[9] Patel KM, Tsui DWY (2015). The translational potential of circulating tumour DNA in oncology. Clin Biochem.

[10] Schwarzenbach H, Hoon DSB, Pantel K (2011). Cell-free nucleic acids as biomarkers in cancer patients. Nat Rev Cancer.

[11] Shapiro B, Chakrabarty M, Cohn EM, Leon SA (1983). Determination of circulating DNA levels in patients with benign or malignant gastrointestinal disease. Cancer.

[12] Shen F, Zheng R-D, Shi J-P, Mi Y-Q, Chen G-F, Hu X, Liu Y-G, Wang X-Y, Pan Q, Chen G-Y, Chen J-N, Xu L, Zhang R-N, Xu L-M, Fan J-G (2015). Impact of skin capsular distance on the performance of controlled attenuation parameter in patients with chronic liver disease. Liver Int..

[13] Boursier J, Zarski J-P, de Lédinghen V, Rousselet M-C, Sturm N, Lebail B, Fouchard-Hubert I, Gallois Y, Oberti F, Bertrais S, Calès P (2013). Determination of reliability criteria for liver stiffness evaluation by transient elastography. Hepatology.

[14] Karlas T, Petroff D, Garnov N, Böhm S, Tenckhoff H, Wittekind C, Wiese M, Schiefke I, Linder N, Schaudinn A, Busse H, Kahn T, Mössner J, Berg T, Tröltzsch M, Keim V, Wiegand J (2014). Non-invasive assessment of hepatic steatosis in patients with NAFLD using controlled attenuation parameter and 1H-MR spectroscopy. PLoS ONE.

[15] Karlas T, Berger J, Garnov N, Lindner F, Busse H, Linder N, Schaudinn A, Relke B, Chakaroun R, Tröltzsch M, Wiegand J, Keim V (2015). Estimating steatosis and fibrosis: comparison of acoustic structure quantification with established techniques. World J Gastroenterol.

[16] Wong VW, Vergniol J, Wong GL, Foucher J, Chan HL, Le Bail B, Choi PC, Kowo M, Chan AW, Merrouche W, Sung JJ, de Lédinghen V (2010). Diagnosis of fibrosis and cirrhosis using liver stiffness measurement in nonalcoholic fatty liver disease. Hepatology.

[17] Breitbach S, Tug S, Helmig S, Zahn D, Kubiak T, Michal M, Gori T, Ehlert T, Beiter T, Simon P (2014). Direct quantification of cell-free, circulating DNA from unpurified plasma. PLoS ONE.

[18] Breitbach S, Sterzing B, Magallanes C, Tug S, Simon P (2014). Direct measurement of cell-free DNA from serially collected capillary plasma during incremental exercise. J Appl Physiol.

[19] Kamel AM, Teama S, Fawzy A, El Deftar M (2016). Plasma DNA integrity index as a potential molecular diagnostic marker for breast cancer. Tumour Biol.

[20] Wang BG, Huang H-Y, Chen Y-C, Bristow RE, Kassauei K, Cheng C-C, Roden R, Sokoll LJ, Chan DW, Shih I-M (2003). Increased plasma DNA integrity in cancer patients. Cancer Res.

[21] Bates D, Mächler M, Bolker B, Walker S (2015). Fitting linear mixed-effects models using lme4. J Stat Softw.

[22] Buzzetti E, Pinzani M, Tsochatzis EA (2016). The multiple-hit pathogenesis of non-alcoholic fatty liver disease (NAFLD). Metabolism..

[23] Luedde T, Kaplowitz N, Schwabe RF (2014). Cell death and cell death responses in liver disease: mechanisms and clinical relevance. Gastroenterology.

[24] Eguchi A, Wree A, Feldstein AE (2014). Biomarkers of liver cell death. J Hepatol.

[25] Guicciardi ME, Malhi H, Mott JL, Gores GJ (2013). Apoptosis and necrosis in the liver. Compr Physiol..

[26] Rodrigues Filho EM, Simon D, Ikuta N, Klovan C, Dannebrock FA, Oliveira de Oliveira C, Regner A (2014). Elevated cell-free plasma DNA level as an independent predictor of mortality in patients with severe traumatic brain injury. J Neurotrauma.

[27] De Vlaminck I, Valantine HA, Snyder TM, Strehl C, Cohen G, Luikart H, Neff NF, Okamoto J, Bernstein D, Weisshaar D, Quake SR, Khush KK (2014). Circulating cell-free DNA enables noninvasive diagnosis of heart transplant rejection. Sci Transl Med..

[28] García Moreira V, Prieto García B, Baltar Martín JM, Ortega Suárez F, Alvarez FV (2009). Cell-free DNA as a noninvasive acute rejection marker in renal transplantation. Clin Chem.

[29] Beck J, Bierau S, Balzer S, Andag R, Kanzow P, Schmitz J, Gaedcke J, Moerer O, Slotta JE, Walson P, Kollmar O, Oellerich M, Schutz E (2013). Digital droplet PCR for rapid quantification of donor DNA in the circulation of transplant recipients as a potential universal biomarker of graft injury. Clin Chem.

[30] Kowdley KV, Belt P, Wilson LA, Yeh MM, Neuschwander-Tetri BA, Chalasani N, Sanyal AJ, Nelson JE (2012). Serum ferritin is an independent predictor of histologic severity and advanced fibrosis in patients with nonalcoholic fatty liver disease. Hepatology.

[31] Kell DB, Pretorius E (2014). Serum ferritin is an important inflammatory disease marker, as it is mainly a leakage product from damaged cells. Metallomics..

[32] Goh GBB, Issa D, Lopez R, Dasarathy S, Dasarathy J, Sargent R, Hawkins C, Pai RK, Yerian L, Khiyami A, Pagadala MR, Sourianarayanane A, Alkhouri N, McCullough AJ (2016). The development of a non-invasive model to predict the presence of non-alcoholic steatohepatitis in patients with non-alcoholic fatty liver disease. J Gastroenterol Hepatol.

[33] McGill MR (2016). The past and present of serum aminotransferases and the future of liver injury biomarkers. EXCLI J..

[34] Nishimoto S, Fukuda D, Higashikuni Y, Tanaka K, Hirata Y, Murata C, Kaneyama J, Sato F, Bando M, Yagi S, Soeki T, Hayashi T, Imoto I, Sakaue H, Shimabukuro M, Sata M (2016). Obesity-induced DNA released from adipocytes stimulates chronic adipose tissue inflammation and insulin resistance. Sci Adv.

[35] Ermakov AV, Konkova MS, Kostyuk SV, Izevskaya VL, Baranova A, Veiko NN (2013). Oxidized extracellular DNA as a stress signal in human cells. Oxid Med Cell Longev..

[36] Garcia-Martinez I, Santoro N, Chen Y, Hoque R, Ouyang X, Caprio S, Shlomchik MJ, Coffman RL, Candia A, Mehal WZ (2016). Hepatocyte mitochondrial DNA drives nonalcoholic steatohepatitis by activation of TLR9. J Clin Invest..

[37] Thomas H (2016). NASH: NASH and TLR9. Nat Rev Gastroenterol Hepatol..

[38] Basnet S, Zhang Z, Liao W, Li S, Li P, Ge H (2016). The prognostic value of circulating cell-free DNA in colorectal cancer: a meta-analysis. J Cancer..

[39] Tamminga S, van Maarle M, Henneman L, Oudejans CBM, Cornel MC, Sistermans EA (2016). Maternal plasma DNA and RNA sequencing for prenatal testing. Adv Clin Chem.

[40] Goossens N, Hoshida Y, Song WM, Jung M, Morel P, Nakagawa S, Zhang B, Frossard J-L, Spahr L, Friedman SL, Negro F, Rubbia-Brandt L, Giostra E (2016). Nonalcoholic Steatohepatitis is associated with increased mortality in obese patients undergoing bariatric surgery. Clin Gastroenterol Hepatol.

[41] Lehmann-Werman R, Neiman D, Zemmour H, Moss J, Magenheim J, Vaknin-Dembinsky A, Rubertsson S, Nellgard B, Blennow K, Zetterberg H (2016). Identification of tissue-specific cell death using methylation patterns of circulating DNA. Proc Natl Acad Sci USA.

[42] Hardy T, Zeybel M, Day CP, Dipper C, Masson S, McPherson S, Henderson E, Tiniakos D, White S, French J, Mann DA, Anstee QM, Mann J (2016). Plasma DNA methylation: a potential biomarker for stratification of liver fibrosis in non-alcoholic fatty liver disease. Gut.

